# The Mediating Effect of Grit in the Relationship Between Middle School Students’ Trust in Their Physical Education Teachers and Health-Promoting Behaviors: Evidence from Korea

**DOI:** 10.3390/healthcare13141650

**Published:** 2025-07-09

**Authors:** Ho-Hyun Song, Wi-Young So, Ji-Heum Park

**Affiliations:** 1 Department of Physical Education, College of Fourth, Korea National University of Education, Cheongju 28173, Republic of Korea; hohyunss@jbedu.kr; 2 Department of Sports Medicine, College of Humanities, Korea National University of Transportation, Chungju-si 27469, Republic of Korea

**Keywords:** grit, health-promoting behaviors, middle school students, structural equation modeling, trust in physical education teachers

## Abstract

**Objectives/Background**: With increasing awareness of the association between physical activity and mental health, promoting youth health has gained prominence. For this, education and support are needed. As psychological school-based factors could be key to affecting this behavior, this study investigates middle school students’ trust in their physical education teachers and their grit, analyzing their effects on health-promoting behaviors that could follow these adolescents through adulthood. **Methods:** Middle school students, aged 12–14, were recruited from three schools in Sejong City, Korea, in May 2025; 420 survey questionnaires were distributed and 390 were returned (response rate: 92.86%). After eliminating those with insincere responses, 369 valid questionnaires (boys = 186, girls = 183) were analyzed. The analysis covered the descriptive statistics, Pearson’s correlation, and structural equation modeling, with grit, trust in physical education teachers, and health-promoting behaviors as variables. **Results:** The correlation analysis verified multicollinearity between trust in physical education teachers (closeness, fairness, teaching method, and physical ability), grit (effort, perseverance, and interest consistency), and health-promoting behaviors (self-actualization, health management, and stress management). A positive significant correlation was found between all subfactors (*p* < 0.05). The research model’s fit was confirmed through several fit indices; specifically, normed χ^2^ = 4.138, goodness-of-fit-index = 0.942, root mean square residual = 0.033, root mean square error of approximation = 0.092, incremental fit index = 0.965, Tucker–Lewis index = 0.947, and comparative fit index = 0.965, and all values were judged acceptable. The standardized coefficients of each latent variable explaining the measurement variables were 0.707 or higher. Therefore, the explanatory power of the measurement variables was also satisfactory; thus, the research model was appropriate and could be used for analysis. The model findings revealed that trust in physical education teachers had a positive effect on student grit (β = 0.505, *p* < 0.001) and that grit had a positive effect on health-promoting behaviors (β = 0.743, *p* < 0.001); however, trust in physical education teachers did not have a direct effect on health-promoting behaviors (statistically insignificant [β = 0.103, *p* > 0.05]). Thus, grit had a mediating effect between trust in physical education teachers and health-promoting behaviors (β = 0.375, *p* < 0.01). **Conclusions:** This study highlights the educational implications for physical education teachers of building trust and strengthening student grit as key factors in achieving sustainable health-promoting behaviors among adolescents.

## 1. Introduction

Today, both teachers and parents recognize the need for education and support that promotes youth health, as the association between physical activity and mental health is well known; namely, as physical activity decreases, mental health issues increase [1]. In general, increased smartphone usage and greater academic burdens have decreased the time that adolescents spend exercising. This is particularly true in Korea, where health and physical fitness levels have decreased and obesity rates have increased. Moreover, the recent COVID-19 pandemic exacerbated this situation [1]. The increasing rate of stress and depression among adolescents in Korea is also concerning, highlighting the need to focus on their psychological health as well [1]. To ensure adolescents’ physical and mental well-being, continuous and systematic health management practices are needed that go beyond simple exercise participation and promote healthy behaviors.

***Adolescent health-promoting behaviors***

Health-promoting behaviors include activities that are part of a lifestyle that maintains or improves a sense of well-being, self-realization, or achievement [2]. An understanding of healthy behaviors improves the ability to control health determinants and achieve well-being [3]. Adolescent behavior in this regard is critical. The National Academies of Sciences, Engineering, and Medicine and others [4] have concluded that developing health-promoting and disease-prevention strategies among adolescents is crucial, as most lifelong health-related habits are formed during this period. Liu et al. [5] reported that unhealthy lifestyles during adolescence were important factors leading to chronic diseases in the future. Moreover, from a psychological perspective, strengthening health-promoting behaviors can help alleviate various types of negative emotions [6]. Physical activity plays an important role in promoting mental health in adolescents and has a positive effect on emotional stability and reducing anxiety, especially through self-esteem [7]. It can also reduce depression and strengthen social bonds [8]. To address these mental issues, peer-led health behavior interventions are attracting attention as effective prevention and intervention strategies [9]. Such interventions also have been used to promote physical activity participation among adolescents [10]. Thus, there are several psychological factors that could influence physical activity and, consequently, adolescent healthy behaviors.

***The role of physical education teachers***

With the increasing awareness of the psychological benefits of physical activity, the role of school physical education has become particularly important. Stellefson et al. [11] argued that health-promoting behaviors can be improved by health education that encourages positive health perceptions among students. In general, school physical education is key to helping adolescents develop balanced physical and mental health. Accordingly, physical education teachers play important roles in supporting students’ holistic growth beyond simple exercises. In addition to challenging students physically, these teachers can contribute to fostering students’ social and emotional stability. Liu et al. [12] reported that feedback from physical education teachers had a positive effect on students’ intention to continue exercising and their participation, mediated by perceived competence and exercise persistence. Viksi and Tilga [13] reported that controlling behavior from physical education (PE) teachers hindered student autonomy, leading to a decrease in participation in physical activities during leisure time.

***The role of trust***

In this context, trust is a key factor in forming, developing, and maintaining the relationship between the student and the PE teacher [14]. Students’ trust in their teachers increases class participation, positive attitudes, and stronger motivation to perform. Sun et al. [15] reported that teacher trust had a positive effect on student learning. Similarly, Sakineh and Ali [16] showed that trust between students and teachers contributed to improving student academic achievement through stronger school interaction and academic motivation. Şirin et al. [17] reported that trust in teachers had a positive effect on class and school participation. Dai [18] argued that positive teacher–student relationships could regulate students’ mental emotions, increase students’ class participation, cultivate students’ learning ability, and promote students’ academic achievement and goals. From the PE perspective, Kim [19] reported that students’ trust in their PE teachers positively affected their intention to continue to exercise. Thus, in this relationship, trust between PE teachers and students positively affected not only attitudes toward physical activities but also students’ health-promoting behaviors.

***The role of grit***

The concept of grit is another factor affecting student success. Grit refers to the ability to persevere until the end of a task without giving up early, despite facing difficulties [20]. Studies have reported an association between grit and academic achievement. For example, Yaure et al. [21] showed that grit could predict the perseverance required to endure physical, mental, and emotional stressors and was positively related to improved academic achievement, which is in line with Allen et al.’s [22] argument. Improving adolescents’ grit may be an important factor in continuous health-promoting behavior as well. Recent studies suggest that grit is closely related not only to academic achievement but also to participation in physical activities and healthy behaviors. Rafiee et al. [23] found that grit had a positive effect on the exercise self-efficacy of athletes. Lee and Hwang [24] reported that the grit of participants in recreational sports affected exercise passion. Gray et al.’s [25] results showed that grit scores among athletes were higher than those among non-athletes, implying that differences in grit may be an important individual characteristic affecting exercise behavior. De La Cruz et al. [26] reported that grit had a positive effect on adults’ readiness to change exercise behavior, particularly through self-efficacy and autonomous motivation. According to Kurita et al. [27], grit was closely related to health-promoting behaviors in general, including physical activity and eating habits, and it was also linked to weight management and the prevention of chronic diseases.

From an adolescent perspective, Liu et al. [28] found that grit suppressed negative behaviors, such as internet addiction in adolescents, and argued that grit could change adolescents’ motivation. Martin et al. [29] reported that grit showed a positive relationship with various health behaviors, such as exercise, sedentary time, and eating habits. Hein et al. [30] reported that grit’s effort persistence led to actual participation in moderate-to-vigorous physical activity through the intention to participate. According to Dai et al. [31], grit was one of the key factors explaining exercise persistence in adolescents, with self-efficacy and self-regulation acting as mediating factors. Given this, grit may be a key factor influencing students’ adoption of health-promoting behaviors.

***Hypothesis development***

Although research on grit is extensive, few studies have examined whether it has a mediating effect on voluntary adolescent behaviors (i.e., health-promoting behaviors). As a mediator, grit could be key to adolescent commitment to physical activity, as well as to health-promoting behaviors. Support from and trust in PE teachers could also be a key factor in strengthening students’ grit, ultimately leading to more voluntary health-promoting behaviors.

**Figure 1:**
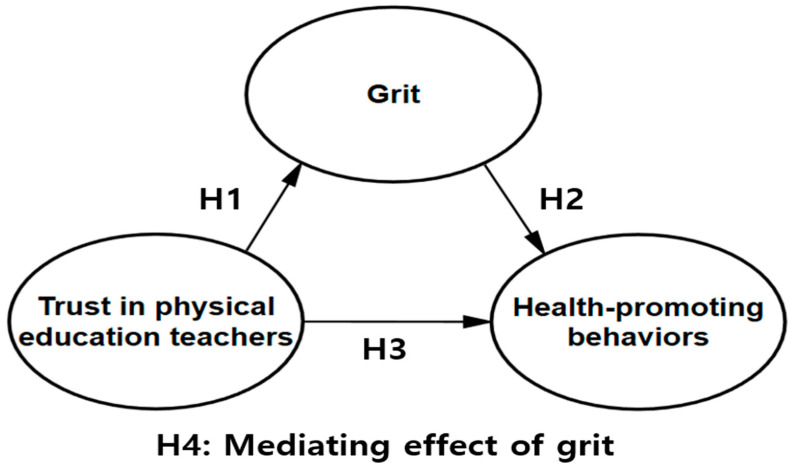
Research model.

To assess the dynamics of these factors, our study looks at middle school students’ trust in PE teachers and their grit to assess the impact of these on health-promoting behaviors, considering grit as a mediator. Thus, we fill a gap in the literature by testing how, through the development of grit as a driver of continuous exercise practice, trust in PE teachers encourages health-promoting behaviors among adolescents. Hence, distinct from previous research, we explore the mediating role of grit in the relationship between PE teacher trust and healthy behaviors. Accordingly, we posited the following hypotheses among middle school students (Figure 1).

**H1:**  *Trust in their PE teachers influences their grit.*

**H2:**  *Grit influences their health-promoting behaviors.*

**H3:**  *Trust in their PE teachers influences their health-promoting behaviors.*

**H4:**  *Grit has a mediating effect in the relationship between their trust in their PE teachers and their health-promoting behaviors.*

We expect our results to provide PE teachers with ways to improve grit and students’ health-promoting behaviors based on trust, clearly presenting a direction for PE going forward.

## 2. Materials and Methods

### 2.1. Design and Procedure

We chose Sejong City in Korea as the setting for our study. This is a new city in Korea with a large concentration of central government agencies. It has a well-established educational infrastructure and a relatively homogeneous educational environment centered on public education. Korea has a nine-year compulsory education system encompassing six years in elementary school and three in middle school. Moreover, relatively uniform nationwide educational content is provided through a national-level curriculum. Since Sejong City is largely a civil servant city, most of the parents would be of similar socio-economic status. As such, we could use non-probability convenience sampling in the city, as the schools’ population characteristics would be relatively homogeneous.

We designed a questionnaire survey that could be distributed to schools in the city, encompassing our study variables (see measures below), and then we analyzed the data through structural equation modeling. To choose the appropriate sample size, we used a Monte Carlo simulation to identify sample requirements for maximum likelihood. The results showed that the minimum sample for maximum likelihood was 200 [32,33,34]. Thus, the sample should be 200 participants or more, if possible. If the sample exceeds 500, maximum likelihood would be too sensitive, and the model fit would be poor [32,33,34]. Therefore, we chose 420 participants as our target sample size.

### 2.2. Participants

After obtaining permission from the school administrations, we distributed 420 surveys to three middle schools in Sejong City from 9 May 2025 to 31 May 2025. For our screening, we included only students 12 to 15 years of age in middle school. There were no other exclusion criteria. We received 390 returned questionnaires (response rate 92.86%), of which 369 were included in our final analysis; 21 questionnaires were excluded for insincere answers. Students were given a small gift for participating.

The study was approved by the Institutional Review Board of the Korea National University of Education (approval number KNUE-202505-SB-0204-01, approval date 8 May 2025) and conducted according to the principles outlined in the Declaration of Helsinki. All participants and their guardians were told the purpose of the study and provided informed written consent.

### 2.3. Measures

The questions we chose for our survey had been verified for reliability and validity in the literature [35,36,37,38,39,40,41]. We then modified and supplemented these according to our objectives. The initial questions covered students’ demographic characteristics: sex, grade, and exercise frequency. The questions on trust in PE teachers that we used had been developed and validated by Park and Park [35] in the Korean language. We also applied a middle school trust in physical education teachers scale developed and validated by Kim et al. [36] in the Korean language. We then modified and supplemented these.

The subfactors under trust in PE teachers comprised four categories: closeness, fairness, teaching method, and physical ability. These were measured with four items for closeness and three items, respectively, for fairness, teaching method, and physical ability, totaling thirteen items. The questions on grit were modified and supplemented as a validated Korean language grit scale based on Ha et al. [37] (previously validated in Duckworth’s [38] original grit scale). The subfactors under grit consisted of two categories: effort perseverance, measured with eight items, and interest consistency, measured with four items. The questions on health-promoting behavior were taken from Walker et al. [39] and validated in the Korean language in the research [40,41]. The subfactors under health-promoting behaviors consisted of three categories: self-actualization (measured with 10 items), health management (measured with 6 items), and stress management (measured with 5 items). All questions were rated on a five-point Likert scale, ranging from one “not at all” to five “very much”.

### 2.4. Data Analysis

We conducted several different analyses of the data. First, we assessed trends and normality using descriptive statistics. We then calculated skewness and kurtosis to confirm the assumption of normality. If skewness is ±3.00 or less and kurtosis is ±8.00 or less, the data meet normality [32,33,34]. Second, we conducted confirmatory factor analysis using the maximum likelihood estimation method and calculated Cronbach’s α to verify the validity and reliability of our measurement tool. To ensure its structural validity, each factor was set as a single-factor model, and confirmatory factor analysis was performed individually. Through this, the factor loadings and fit indices between items were examined, and the appropriateness of the measurement structure for each scale was checked in advance before the overall structural model analysis. As a criterion for suitability, normed χ^2^ is acceptable if it is 3.00 or less at a strict level and 5.00 or less at a lenient level [32,33,34]. The goodness-of-fit-index (GFI) is considered sufficient if it is 0.900 or higher; the root mean square residual (RMR) is considered acceptable if it is 0.050 or lower; the root mean square error of approximation (RMSEA) is average if it is 0.100 or lower; and the incremental fit index (IFI), the Tucker–Lewis index (TLI), and the comparative fit index (CFI) are acceptable if they are 0.900 or higher [32,33,34]. Cronbach’s α of 0.600 or higher represents appropriate internal consistency. Third, we ran Pearson’s correlation analysis to assess multicollinearity between variables and conducted path analysis of the structural equation model to analyze the relationship between variables. Finally, we ran bootstrapping 2000 times to verify the mediation effect at 95% confidence using a bias correction method. We used SPSS (version 29.0; IBM Corp., Armonk, NY, USA) and AMOS (version 29.0; IBM Corp., Armonk, NY, USA) for all of our statistical analyses, with statistical significance set at *p* < 0.05.

## 3. Results

### 3.1. Participant Characteristics

The participant characteristics are shown in Table 1. Our sample was equally representative of boys and girls and the three grades (7th–9th). The age range was from 12 to 15 years old. Approximately one-third of the students exercised three times a week, the highest frequency.

### 3.2. Descriptive Statistics of Each Variable

The descriptive statistics of the variables are shown in Table 2. The mean of the variables was between 3.259 and 4.178, and the standard deviation was between 0.717 and 0.875. The skewness was between −1.165 and 0.294, and the kurtosis was between −0.251 and 1.962, confirming a normal distribution.

### 3.3. Confirmatory Factor Analysis

To assure validity, experts in the field (one professor and two doctors specializing in sports education) reviewed the content validity in advance. As stated, we also conducted confirmatory factor analysis using the maximum likelihood estimation method and Cronbach’s α to verify internal consistency and reliability of the items. We found that for trust in PE teachers, the fit indices were χ^2^ = 145.986, GFI = 0.945, RMR = 0.024, RMSEA = 0.063, IFI = 0.977, TLI = 0.969, and CFI = 0.976. Cronbach’s α was 0.907 for closeness, 0.851 for fairness, 0.884 for teaching method, and 0.850 for physical ability, verifying reliability.

We found that for grit, the fit indices were χ^2^ = 304.53, GFI = 0.858, RMR = 0.062, RMSEA = 0.114, IFI = 0.915, TLI = 0.894, and CFI = 0.915. Although RMSEA exceeded the goodness-of-fit criterion, the index is not an absolute value. Thus, we interpreted this as an approximate and acceptable value [32,33,34]. Cronbach’s α was 0.916 for effort perseverance and 0.783 for interest consistency, again confirming reliability.

We also found that for health-promoting behavior, the fit indices were χ^2^ = 503.692, GFI = 0.882, RMR = 0.066, RMSEA = 0.069, IFI = 0.913, TLI = 0.899, and CFI = 0.912, and Cronbach’s α was 0.908 for self-actualization, 0.773 for health management, and 0.811 for stress management, again confirming reliability. The confirmatory factor analysis results are shown in Table 3.

### 3.4. Correlation Analysis

We conducted a correlation analysis to verify multicollinearity among trust in PE teachers (closeness, fairness, teaching method, and physical ability), grit (effort perseverance and interest consistency), and health-promoting behaviors (self-actualization, health management, and stress management). We found a positive and significant correlation among all subfactors. The subfactors with the highest values were teaching method and fairness (0.775), and those with the lowest were health management and physical ability (0.106). In addition, the results of the correlation showed that all values were less than 0.800, indicating no problem with multicollinearity. The specifics are shown in Table 4.

### 3.5. Goodness-of-Fit Index and Parameter Estimates

We confirmed our research model’s fit through the fit indices. Specifically, normed χ^2^ = 4.138, GFI = 0.942, RMR = 0.033, RMSEA = 0.092, IFI = 0.965, TLI = 0.947, and CFI = 0.965. The results of the goodness-of-fit test were judged to be acceptable for all values (see Table 5). In addition, as shown in Table 6, the standardized coefficients of each latent variable explaining the measurement variables were 0.707 or higher. Therefore, the explanatory power of the measurement variables was also satisfactory; thus, the research model was appropriate and could be used for analysis.

### 3.6. Verification of Causal Relationship

**Figure 2:**
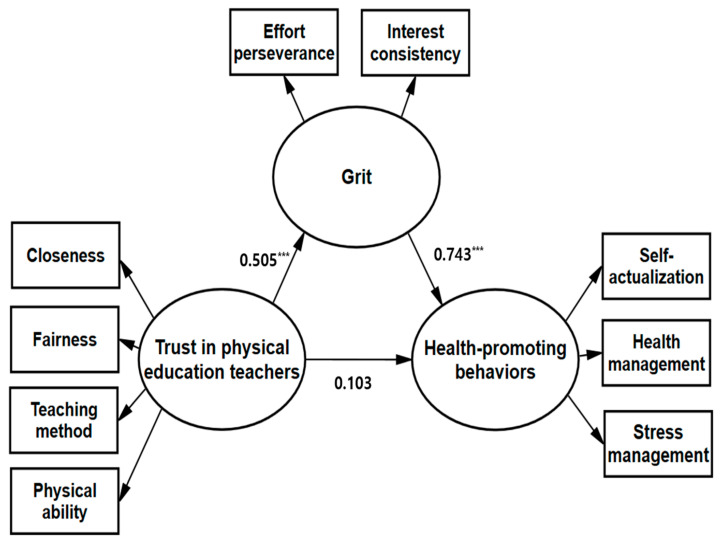
Standardized estimates in the structural equation model (*** *p* < 0.001).

We were able to verify the causal relationships among trust in PE teachers, grit, and health-promoting behaviors. First, in the relationship between trust in PE teachers and grit, the standardized coefficient (β) was 0.505 and the significance (critical ratio) was 8.966, showing statistical significance (*p* < 0.001). Second, in the relationship between grit and health-promoting behaviors, β was 0.743 and the significance (critical ratio) was 11.701, again statistically significant (*p* < 0.001). Third, however, in the relationship between trust in PE teachers and health-promoting behaviors, β was 0.103 and the significance (critical ratio) was 1.961; thus, they were statistically insignificant. Ultimately, the indirect effect of trust in PE teachers on health-promoting behavior through grit was statistically significant at 0.375 (*p* < 0.01). Since trust in physical education teachers did not directly affect health-promoting behaviors, we can discern that grit had a complete mediating effect in the relationship between trust in PE teachers and health-promoting behaviors. The specific results are shown in Table 7 and Table 8 and Figure 2.

## 4. Discussion

Our study aim was to analyze the effects of middle school students’ trust in their PE teachers on grit and their voluntary health-promoting behaviors; we also aimed to identify whether grit had a mediating effect. First, we found that trust in their PE teachers had a positive effect on their grit, supporting Hypothesis 1. This is consistent with the results in previous studies indicating that teacher trust is a basic element in effective student education [42] and that students’ perceptions of teachers they have an emotional connection with improve grit [43]. This result is also consistent with studies indicating that teachers’ attitudes toward students in high school PE classes have a significant effect on students’ grit [44]. Yoo and Park [45] state that when teachers and students form a positive relationship, it enhances students’ inner capabilities and enables continuous commitment to long-term goals.

The implication is that as PE teachers become closer and appear fairer to students, alongside effective teaching methods, student grit is likely to increase. Additionally, it suggests that when a trusting relationship is formed between PE teachers and students, students are likely to challenge themselves more in physical education class assignments, which can also engender grit. Furthermore, grit had a positive effect on the health-promoting behaviors we measured, supporting Hypothesis 2. This is consistent with the results showing that students with high grit tend to exercise consistently and maintain healthy lifestyles. Bae et al. [46] reported that the higher the students’ grit, the higher the scores for exercise and life satisfaction. Stamatis et al. [47] similarly found that grit was an important factor in predicting high- and moderate-intensity exercise. Rutberg et al. [48] argued that creating grit could be the ultimate goal for achieving sustainable student physical activity. Grit was also positively associated with physical activity and leisure time physical activity [49]. Individuals with higher levels of grit reported healthier eating and sleeping behaviors than individuals with lower levels of grit [50].

Gorin et al. [51] concluded that grit played an important role in healthy habits and had a positive effect on exercise persistence. In other words, grit or the consistent effort toward achieving a goal can have a positive effect not only on physical health but also on improving lifestyle habits. These results suggest that PE should include educational approaches to supporting healthy lifestyle habits beyond simple exercise skills.

However, we found that trust in PE teachers had no significant direct effect on voluntary health-promoting behaviors in adolescents, therefore rejecting Hypothesis 3. This result contrasts with previous findings, which indicate that the higher the level of trust in PE teachers, the higher the intention to participate in sports [52]. However, our results suggest that the trust relationship between teachers and students may indirectly affect health-promoting behaviors through students’ psychological factors, namely, grit. Thus, there may be limitations in improving students’ healthy behavior simply by increasing trust in PE teachers. Therefore, improving trust through teaching and learning strategies should be supplemented by developing student grit.

Ultimately, we found that grit mediated the relationship between trust in PE teachers and health-promoting behaviors, supporting Hypothesis 4. This is consistent with studies that point to a high positive correlation among trust in coaches, grit, and the continued exercise intention of college soccer players [53]. This also suggests that trust in PE teachers influences the development of healthy lifestyle habits by strengthening student grit, rather than by directly influencing healthy behavior. In other words, the greater the students’ trust in their PE teachers, the more they perform physical activities based on perseverance and patience; this, then, engenders health-promoting behaviors. As such, PE teachers play the role of psychological supporter; someone who develops student perseverance and patience rather than just offering technical instruction.

In sum, we found that trust in PE teachers was an important factor in strengthening students’ grit, with grit playing an important role in the development of voluntary health-promoting behaviors. As stated above, for this reason, PE teachers should build trusting relationships with their students and use various teaching and learning strategies to enhance their grit. Studies have shown that positive emotions and hope can enhance grit [54] and that encouraging intentional tasks can promote the development of grit [55]. Furthermore, continuous sports participation during adolescence has a positive effect on increasing grit in adulthood [56].

According to Nho and Chae [57], continuous participation in lifestyle intervention is effective in improving students’ health-promoting behaviors. Kamran et al. [58] argued that meaningful improvements in behavior and health promotion can be achieved through planned and targeted systematic student education. In the long term, student grit needs to improve in PE classes, and educational experiences need to be practically relevant to students’ present and future lives [59]. Teachers need to establish and promote positive teacher–student relationships and improve students’ learning outcomes by being listeners of mental health issues, leaders of academic learning, and supporters of creative thinking [18]. The study by Ria and Eliasa [60] confirmed that open communication and inclusive teaching methods contributed to improving learning motivation and academic achievement and reducing students’ problem behaviors. Ultimately, teacher education and training policies need to be developed to ensure high-quality teacher–student interactions [60].

Based on our analysis, we offer the following recommendations: PE teachers should develop close relationships with their students while exhibiting fairness towards all students; highlight the benefits of continuous sports participation and the importance of healthy lifestyle habits; and establish a foundation for regular exercise habits through their classes. Finally, they should focus on increasing student grit by creating long-term goals, achievable and challenging tasks, and successful student experiences.

Although our study contributed to the literature by confirming the importance of grit in the relationship between trust in PE teachers and adolescent health-promoting behaviors, it also had some limitations. First, since we limited the study participants to middle school students in one region of Korea (Sejong City), it may be difficult to generalize the findings to all Korean students. Second, we did not investigate the cultural context, such as whether collectivist norms in Korea influence trust or grit differently than they do in Western populations. Third, we used a self-reported questionnaire, which may induce bias in the results. Fourth, the fact that validity and reliability were not examined together in an integrated dimension, including all factors, may also be a limitation. Fifth, we only considered a limited number of health-promoting behaviors and excluded a discussion of unhealthy behaviors, such as smoking and drinking. Sixth, a sample size of 369 participants could be considered too limiting. Future research could consider other regions, which would expand the scope of the study population. It could also explore mechanisms for improving trust in PE teachers and grit, as well as PE programs for forming healthy lifestyle habits.

## 5. Conclusions

Our study identified three key results: trust in PE teachers has a positive effect on grit; grit has a positive effect on health-promoting behaviors; and, as trust in PE teachers does not have a significant direct effect on health-promoting behavior, grit has a significant mediating effect in the relationship between trust in PE teachers and health-promoting behaviors. These results underscore the role of grit as a key psychological factor affecting health-promoting behavior among adolescents in more than one way. Thus, it is important to strengthen student trust in PE teachers as well as establish a teaching and learning method that improves grit. The ability to improve grit could have positive, long-term implications for student health in the future.

## Figures and Tables

**Table 1:** Participant characteristics (*n* = 369).

Characteristic	Categories	Frequency (n)	Percentage (%)
Sex	Male	186	50.41
	Female	183	49.59
	Total	369	100.00
Grade	7th	136	36.86
	8th	101	27.37
	9th	132	35.77
	Total	369	100.00
Exercise frequency	1 time/week	62	16.80
	2 times/week	50	13.55
	3 times/week	118	31.97
	4 times/week	42	11.38
	5 times/week	63	17.07
	6 times/week	21	5.69
	7 times/week	13	3.52
	Total	369	100.00

**Table 2:** Descriptive statistics of each variable.

Variables	Factor	Average	Standard Deviation	Skewness	Kurtosis
Trust in physical education teachers	Closeness	4.083	0.813	−0.880	0.721
	Fairness	3.866	0.875	−0.696	0.400
	Teaching method	3.963	0.834	−0.717	0.763
	Physical ability	4.178	0.773	−1.165	1.962
Grit	Effort perseverance	3.814	0.840	−0.293	−0.251
	Interest consistency	3.538	0.725	0.294	−0.182
Health-promoting behaviors	Self-actualization	3.865	0.717	−0.322	0.075
	Health management	3.259	0.816	−0.020	0.192
	Stress management	3.673	0.800	−0.207	0.098

**Table 3:** Confirmatory factor analysis results.

Variables	Sub-Variables	B	β	Standard Error	Critical Ratio	Cronbach’s α	Model Fit
Trust in physical education teachers	Closeness 1	0.973	0.853	0.046	21.188 ***	0.907	χ^2^ = 145.986 df = 59 GFI = 0.945 RMR = 0.024 RMSEA = 0.063 IFI = 0.977 TLI = 0.969 CFI = 0.976
	Closeness 2	1.200	0.861	0.056	21.540 ***		
	Closeness 3	0.890	0.815	0.045	19.582 ***		
	Closeness 4	1.000	0.861	-	-		
	Fairness 1	0.787	0.759	0.049	15.979 ***	0.851	
	Fairness 2	1.012	0.856	0.054	18.661 ***		
	Fairness 3	1.000	0.819	-	-		
	Teaching method 1	1.035	0.851	0.050	20.878 ***	0.885	
	Teaching method 2	1.078	0.840	0.053	20.417 ***		
	Teaching method 3	1.000	0.860	-	-		
	Physical ability 1	1.102	0.862	0.066	16.729 ***	0.850	
	Physical ability 2	1.021	0.812	0.065	15.748 ***		
	Physical ability 3	1.000	0.755	-	-		
Grit	Effort perseverance 1	0.941	0.841	0.055	17.014 ***	0.916	χ^2^ = 304.53 df = 53 GFI = 0.858 RMR = 0.062 RMSEA = 0.114 IFI = 0.915 TLI = 0.894 CFI = 0.915
	Effort perseverance 2	0.949	0.835	0.056	16.870 ***		
	Effort perseverance 3	1.008	0.841	0.059	17.000 ***		
	Effort perseverance 4	1.010	0.823	0.061	16.590 ***		
	Effort perseverance 5	0.919	0.834	0.055	16.854 ***		
	Effort perseverance 6	0.906	0.735	0.062	14.556 ***		
	Effort perseverance 7	0.869	0.720	0.061	14.237 ***		
	Effort perseverance 8	1.000	0.754	-	-		
	Interest consistency 1	1.175	0.781	0.086	13.682 ***	0.783	
	Interest consistency 2	1.241	0.820	0.087	14.217 ***		
	Interest consistency 3	1.228	0.793	0.089	13.849 ***		
	Interest consistency 4	1.000	0.722	-	-		
Health-promoting behaviors	Self-actualization 1	0.775	0.668	0.063	12.384 ***	0.908	χ^2^ = 503.692 df = 183 GFI = 0.882 RMR = 0.066 RMSEA = 0.069 IFI = 0.913 TLI = 0.899 CFI = 0.912
	Self-actualization 2	0.723	0.670	0.058	12.430 ***		
	Self-actualization 3	0.695	0.552	0.068	10.199 ***		
	Self-actualization 4	0.797	0.654	0.066	12.120 ***		
	Self-actualization 5	0.950	0.779	0.065	14.508 ***		
	Self-actualization 6	0.974	0.752	0.070	13.992 ***		
	Self-actualization 7	1.028	0.744	0.064	16.035 ***		
	Self-actualization 8	1.000	0.725	-	-		
	Self-actualization 9	0.867	0.677	0.069	12.568 ***		
	Self-actualization 10	1.043	0.775	0.072	14.428 ***		
	Health management 1	1.472	0.644	0.215	6.833 ***	0.773	
	Health management 2	1.498	0.656	0.218	6.876 ***		
	Health management 3	1.731	0.783	0.239	7.246 ***		
	Health management 4	1.000	0.403	-	-		
	Health management 5	1.532	0.728	0.216	7.107 ***		
	Health management 6	1.124	0.484	0.186	6.047 ***		
	Stress management 1	0.942	0.746	0.066	14.190 ***	0.811	
	Stress management 2	0.714	0.698	0.054	13.194 ***		
	Stress management 3	0.861	0.677	0.067	12.767 ***		
	Stress management 4	0.734	0.552	0.072	10.233 ***		
	Stress management 5	1.000	0.777	-	-		

Notes: *** *p* < 0.001; assessed using confirmatory factor analysis and Cronbach’s α. GFI, goodness-of-fit-index; RMR, root mean square residual; RMSEA, root mean square error of approximation; IFI, incremental fit index; TLI, Tucker–Lewis index; CFI, comparative fit index.

**Table 4:** Correlation analysis results.

	1	2	3	4	5	6	7	8	9
1	1.000								
2	0.692 ***	1.000							
3	0.760 ***	0.775 ***	1.000						
4	0.752 ***	0.655 ***	0.735 ***	1.000					
5	0.367 ***	0.439 ***	0.406 ***	0.248 ***	1.000				
6	0.368 ***	0.423 ***	0.435 ***	0.249 ***	0.711 ***	1.000			
7	0.360 ***	0.442 ***	0.385 ***	0.253 ***	0.666 ***	0.539 ***	1.000		
8	0.216 ***	0.332 ***	0.271 ***	0.106 *	0.516 ***	0.513 ***	0.620 ***	1.000	
9	0.334 ***	0.431 ***	0.393 ***	0.278 ***	0.530 ***	0.455 ***	0.714 ***	0.581 ***	1.000

Notes: * *p* < 0.05, *** *p* < 0.001; assessed using Pearson correlation analysis. 1, closeness; 2, fairness; 3, teaching method; 4, physical ability; 5, effort perseverance; 6, interest consistency; 7, self-actualization; 8, health management; 9, stress management.

**Table 5:** Goodness-of-fit indices of the research model.

	Normed χ^2^	GFI	RMR	RMSEA	IFI	TLI	CFI
Goodness-of-fit index	4.138 (χ^2^ = 99.319, df = 24)	0.942	0.033	0.092	0.965	0.947	0.965
Criteria of model fit	<5.000	≥0.900	≤0.050	≤0.100	≥0.900	≥0.900	≥0.900

GFI, goodness-of-fit-index; RMR, root mean square residual; RMSEA, root mean square error of approximation; IFI, incremental fit index; TLI, Tucker–Lewis index; CFI, comparative fit index.

**Table 6:** Parameter estimates of the research model.

**Variables**	**Factor**	**B**	**β**	**Standard Error**	**Critical Ratio**
Trust in physical education teachers	Closeness	1.101	0.853	0.057	19.175 ***
	Fairness	1.165	0.838	0.062	18.689 ***
	Teaching method	1.202	0.907	0.058	20.812 ***
	Physical ability	1.000	0.815	-	-
Grit	Effort perseverance	1.000	0.901	-	-
	Interest consistency	0.756	0.789	0.047	16.037 ***
Health-promoting behaviors	Self-actualization	1.000	0.897	-	-
	Health management	0.898	0.707	0.059	15.156 ***
	Stress management	0.984	0.791	0.056	17.605 ***

Notes: *** *p* < 0.001; assessed using structural equation modeling.

**Table 7:** Estimate of the direct paths.

Path				B	*β*	Standard Error	Critical Ratio	Hypothesis
H1	Trust in physical education teachers	→	Grit	0.607	0.505	0.068	8.966 ***	Supported
H2	Grit	→	Health-promoting behaviors	0.632	0.743	0.054	11.701 ***	Supported
H3	Trust in physical education teachers	→	Health-promoting behaviors	0.105	0.103	0.054	1.961	Rejected

*** *p* < 0.001; assessed using structural equation modeling.

**Table 8:** Estimate of the indirect path.

Indirect Path						Estimate	Standard Error	95% Confidence Interval		Hypothesis
								Lower	Upper	
H4	Physical education teacher trust	→	Grit	→	Health-promoting behaviors	0.384 **	0.052	0.279	0.489	Supported

** *p* < 0.01; assessed using structural equation modeling.

## Data Availability

The data supporting the findings of this study are available from the corresponding author upon request.
